# Abstracts and Gleanings

**Published:** 1879-10-20

**Authors:** 


### ABSTRACTS AND GLEANINGS.

**Galvanic Current in Exophthalmic Goitre.**—Dr. Rockwell, in N. Y. Med. Record, reports the following cases:

*Case* 1. The patient, John L——, was a pale, slim man, aged 29, and by occupation a compositor. The three cardinal symptoms of the disease, viz.: exophthalmus, swelling of the thyroid gland, and palpitation, were present in a marked degree, and in addition there was a want of harmony between the movements of the upper eyelid and eyeball, a phenomenon first observed by Von Graefe, and by him regarded as pathognomonic.

The history and antecedents of the case are as follows: The mother, now deceased, suffered from epileptic seizures from the earliest remembrance of our patient, while an older sister was the victim of frequent and severe attacks of hysteria. The father had been intemperate, and died of delirium tremens. It would thus seem that we had in this history ground for a belief in the importance of the relation of hereditary influences to these conditions.

The health of the patient up to his twenty-fifth year had been uniformly good, and the only evidence of a neurotic predisposition was an occasional and unexplainable tendency to insomnia for a year or so previous to the first symptoms of his disease. I first saw the man July 6, 1879.

During the summer of 1878 he observed a slight swelling of the thyroid; very soon after, considerable palpitation; and later still, protrusion of the eyeballs. It is to be noted in passing that the order of the onset of the symptoms is unusual, the thyroid enlargement usually being second in order of development instead of first.

On my first examination I found the gland enlarged to about the size of the fist of a child of ten years, the pulse beating in frequency 125 to the minute, while the protrusion of the globe of the eye was as great as in any case I have seen.

By subsequent examination I found that the pulse ranged from 110 to 130. On three different occasions, when the axillary thermometer was used, it marked 100°, 101°, 100.4°. The appetite of the patient was poor, his secretions disordered, and his general strength impaired. After some preliminary medication of a corrective nature, I gave the ordinary prescription of quinine and iron, and at the same time began the application of the galvanic current.

My method of application was as follows: Placing the cathode over the cilio-spinal center, and the anode in the auriculo-maxillary fossa, I gradually drew the latter, after a few moments of stabile treatment, along the inner border of the sterno-cleido muscle, to its lower end. Removing now the anode to the position occupied by the cathode, and the cathode to the pit of the stomach, I would continue the treatment with a greatly increased strength of current for some moments longer. My ordinary measurement of current strength is by the "shunt" galvanometer of the long coil variety.

During the first portion of the operation described, the current was sufficiently strong to deflect the needle 15°; during the last half of the operation, the deflection was increased to 30°. In other words, as a freshly charged zinc-carbon battery of regulation strength deflects the needle about 1° for every cell, a current strength equivalent to fifteen and thirty fresh cells was used. In point of fact, however, as some deterioration in activity through previous using had occurred, from twenty to forty cells were employed. The result of this treatment were immediate and of the most positive character.

The force and rapidity of the heart-beats were greatly modified, and, accompanying, or rather following, by a week or ten days, the subsidence of the violent palpitation, there was a very noticeable decrease of the exophthalmus. A decrease in the size of the thyroid was not observed until some days after, and disappeared with less rapidity than the other symptoms. In order to hasten the cure, I very carefully performed electrolysis on two occasions, and with evident benefit.

At the date of writing, August 20th, after having received fifteen (15) applications, the patient has, so far as relates to the palpitation and exophthalmus, entirely recovered.

The goitre has decreased in size fully two-thirds, and is quite hard and firm, a change which is to be attributed, in all probability, to a hyperplasia of the glandular tissue taking the place of the dilated vessels.

From this time forward, the important point in this somewhat remarkable case will relate to the degree of permanency of the apparent recovery.

*Case* 2. Mrs. G——, aged about 40, came to me for the relief of an exophthalmic goitre, May 3, 1876. The eyes were much protruded, the thyroid prominent, and the cardiac palpitation violent. The average frequency of the pulse was about 115, but on various occasions I found that it was beating at the rate of 140 to the minute. The patient was annoyed by profuse bilateral perspiration, the pupils were enlarged, and vertigo was a frequent symptom. The appetite was generally good, but she complained of much nausea.

She referred to one other symptom, which, if related to the disease as an effect, as it would seem, was quite new to me. Subsequent to the development of the three cardinal symptoms, which occurred in the following order: Palpitation, thyroid enlargement, exophthalmus, a swelling appeared near the pit of the stomach, which in size and vigor of pulsation was more marked than the goitre. I may remark that Dr. S. S. Purple, of New York, had attended the patient in several confinements, and was cognizant of the disease in question.

Dr. Purple informed me that she had suffered much from malarial poison, to which, together with the effects of a labor of some severity shortly previous, might possibly be attributed the symptoms in question. The first signs of the disease were manifest some three years before she came under my observation.

I administered to this patient seventeen applications, ten of which were with the galvanic current, locally applied, while seven were with the faradic current, and were more general in their nature. Amelioration followed very quickly, and at this date the only symptoms of the disease is an almost imperceptible swelling of the thyroid, and, in a modified form, a tendency to occasional cardiac palpitations.

**Appearance of the Tongue in Diseases.**—The appearance of the tongue will tell us, 1st.—The condition of the digestive organs. 2d. Function of nutrition and assimilation. 3d. The condition of the blood. All of which we want to know from every patient before we can make, in many cases, a rational or scientific prescription.

Some of the peculiar appearances of the tongue in numerous diseases are the broad, white-coated, pallid tongue; or the deep red sleek tongue. Then we have the pinched, shrunken, elongated, full, yellow-furred, violet, dark brown tongue, with many other varieties.

We shall now attempt to describe all the above appearances, as found in various diseases, but only call attention to the first two forms:

1. The broad, pallid, white-furred tongue, whether found in acute or chronic disease, and even in the same disease at different stages, it matters not, always indicates the want of the alkaline elements of the body. The patient that has such a tongue never wants an acid drink, because his system is already acid, whether the acid is in the stomach or blood it matters not. In such cases, with our usual prescription we must give alkalies, such as sulphate or bicarbonate of soda, in order to neutralize the acid. Then and not till then, will our remedies act kindly and much more promptly, and the patient improve more rapidly. Now, if the acids are prescribed when the tongue is broad and pallid, the system being full of acids already, it will be seen at once that no improvement could or should be expected. I remember before I was aware of this rule, I could not see why my remedies did not improve the patient; but now I give the proper alkali, removing the acid, then the remedies act more promptly, the tongue changes to a better appearance, and the patient improves correspondingly. The acid condition of the system is probably more often found in acute than in chronic diseases; at least, that is my observation. It needs but a trial to test this principle; I have never known it to fail. I do not pretend to explain why the tongue looks thus when there is a superabundance of acid in the system.

2. The deep, sleek tongue, with a slight coat at the base, indicates just the opposite condition; here the system is in an alkaline state, no matter in what form of disease. Then some kind of acids must be given to neutralize the superabundance of the alkali before other remedial agents will or ever can have the proper effect. All persons that have this peculiar tongue desire acid drinks. This tongue is often, (though not always) seen in erysipelas, typhoid fever, and many other diseases, where the tr. chloride of iron or muriatic acid will be the leading remedies. But if the system was in an acid condition, known by the broad, pallid tongue, those remedies would do more harm than good. Now I do not wish to inculcate the idea that alkalies or acids will cure any disease, but the above appearances of the tongue are indicative of specific physical conditions, and those remedies are adjuvants, paving the way and assisting specific remedies.

The other peculiar appearances of the tongue above-named each have a significance, and one very important to learn; not only being a material element in making a diagnosis and prognosis, but are also indicative of treatment. Many of our best remedies fail in chronic diseases just for the want of this simple knowledge, and this is one reason why remedies will remove the disease in one patient and fail in another, though they may have the same disease and the same remedies. Take rheumatism: sometimes an alkaline treatment will cure. Failure often lies in the physician, not in the remedies. The grand secret is to know which of the two is demanded for the particular form of the disease.—*Dr. Henning in Country Prac.*

**Memoranda for the Disinfection of Yellow Fever.**—The following rules have been published by the National Board of Health:

1. It is prudent to assume that the essential cause of yellow fever is what may, for conciseness, be called a "germ," that is, something which is capable of growth and propagation outside the living human body; that this germ flourishes especially in decaying organic matter or filth, and that disinfection must have reference both to the germ and to that in or on which it flourishes.

2. Disinfection, when used in a place not disinfected, for the purpose of rendering filth or foul soils, waters, etc., incapable of propagating diseased germs, is a poor substitute for cleanliness, and is mainly useful to make the process of cleansing odorless and harmless. The best disinfectants for this purpose are sulphate of iron, carbolic acid, fresh quick lime, fresh charcoal powder, chloride of zinc, chloride of aluminum, and permanganate of potash.

3. The two great difficulties in destroying the vitality of the germ of yellow fever are, first, to bring the disinfecting agent into actual contact with the germ; and, second, to avoid injuring or destroying other things which should be preserved.

4. When the germ of yellow fever is dry, or partially dried, no gaseous disinfectant can be relied on to destroy it. It must either be moistened or subjected to a dry heat of not less than 250 degrees Fahrenheit to obtain security.

5. In disinfecting or destroying infected bed clothing, bedding, or movable articles, move them as little as possible while dry. Before disturbing them have them thoroughly moistened, either with a chemical disinfecting solution or with boiling water, in order to prevent the diffusion of dried germs in the air in the form of dust.

6. The best method of disinfecting rooms, buildings, ships, etc., is still doubtful, owing to the difficulty of destroying the vitality of dried germs.

The Board proposes to have this subject carefully investigated, and in the meantime advises thorough scrubbing and moist cleansing, to be followed by the fumes of burning sulphur, at the rate of 18 ounces per 1,000 cubic feet of space to be disinfected.

The sulphur should be broken in small pieces, burned over vessels containing water or sand, which vessels should be distributed in the closed space to be disinfected at the rate of one to each 100 square feet of area of floor.

7. No patented compound known to the Board is superior, as a disinfectant, to the agents above mentioned, and none is so cheap. Some of these patent disinfectants are good deodorants, but the removal of an unpleasant odor is no proof that true disinfection has been accomplished.

8. In districts where yellow fever prevailed last year the following precautionary measures should be taken:

(*a*) Textile fabrics of every description which were exposed to yellow fever infection during the year 1878 and which have remained packed or boxed in a closed space since such exposure, should not be opened or unrolled, but should either be burned or placed in boiling water for half an hour or more, or in suitable heated ovens, or disinfected, according to the nature and value of the individual article or articles.

(*b*) Every house or room in which cases of yellow fever occurred in the year 1878, and since that time have remained unoccupied, should not be opened for occupation until they have been thoroughly cleansed and disinfected by persons acclimated to yellow fever.

(*c*) Every privy, vault, under-ground water-cistern, dry well, or closed cellar, connected with a house in which yellow fever existed last year, and which may not have been opened since that date, should not be reopened, but if possible should be covered with several feet of earth.

(*d*) Every suspicious case of sickness should be at once isolated, and every possible precaution taken to prevent infection by providing attendants who have had the disease, and thorough disinfection of all discharges from the sick. If the disease prove to be yellow fever, all articles of clothing and bedding used about the sick should be burned, the house should be vacated, and every room tightly closed and fumigated with burning sulphur.—*Medical and Surgical Reporter.*

**Martin's Bandage in Chronic Ulcers.**—The treatment of chronic ulcers of the leg in out-patient hospital practice has not hitherto in my experience been attended with great success. It has often consisted in the prescribing of some lotion or ointment, and in the administration of good advice which the patient has been unable to follow. It is an easy thing to tell a patient to go to bed for a month or two, but unfortunately a man who has to work for his family, or a woman who must support herself, often finds that rest is the last thing possible; if they wish to be admitted into a hospital they are told that "ulcerated legs of long standing" are according to law not admissible, and so they go on month after month and year after year with an unhealed leg which is a source of discomfort to themselves and of annoyance to their friends.

Since the commencement of the present year I have used with excellent results the solid rubber bandages introduced by Dr. Martin, of New York. The bandage has been put on next the skin without any other dressing: the patient has been instructed (1) to remove the bandage on going to bed, (2) to wash the leg with warm water and soap, (3) to sponge the ulcer with carbolic acid lotion and keep this applied all night, (4) to have the bandage well washed and hung up unfolded during the night, (5) to again wash the leg in the morning, and (6) to apply the bandage before leaving bed.

The following cases are fairly typical:

1. T. N,, æt. 60; small ulcer, size of half-a-crown, which had been unhealed for six months. The skin over the whole leg is red and shiny; he has been suffering on and off for twenty years; cured in a fortnight, though similar ulcers had hitherto always taken six months to heal: when offered the price of the bandage in return for it said "he would not take a pound (five dollars) for it."

2. H. R., æt. 56; leg ulcerated for two years; the skin is hard and callous and ulcerated in all directions, there being numerous ulcers varying from one to three inches in diameter; has not been able to walk in six months. The application of the bandage gave immediate relief; cure in ten weeks.

3. E. R., æt. 52; ulcer of leg eight years, callous edges which are somewhat inflamed, measure three by two inches. The bandage caused pain for the first week, but since then has been quite comfortable; after two months' treatment the ulcer is nearly healed.

It is unnecessary to multiply cases; of about twenty cases treated in this manner two only have been unrelieved. In both cases the want of success was due to the fact that the ulcers were iuflamed and painful when the bandage was applied; the application much increased the pain and necessitated the discontinuance of the treatment. If applied in suitable *chronic* cases the bandage is more efficacious than any other method of treatment It is, I think, a most valuable addition to the appliances used in minor surgery.—*Dr. McGill in the Practitioner.*

**Habitual Constipation—Its Cure.**—Habitual constipation is a functional inactivity of the bowel which is commonly induced, by and after years of neglect, on the part of the individual to obey the simple daily call of nature. A gradual and ever-increasing distention of the large intestine takes place from the frequent and constant accumulation of fecal matter, which has a paralyzing effect on its contractile power, and still further to produce long retention of that which has become poisonous in its nature. The blood thus becomes deteriorated, and the several organs (vital and other) of the body ultimately suffer. For this very painful and uncomfortable state of things I profess to have found a very simple and very effectual remedy.

The treatment is as follows: The person so suffering should make daily use of an enema composed of that most useful colloid substance of liquid called glycerine, and tepid water, in the proportion of one tablespoonful of the former to half a pint of the latter. This quantity —in some cases even half this quantity is sufficient for the purpose— must be injected into the bowel every day during some leisure hour, the evening being the most convenient as well as the most suitable time for such an operation. The enema at each time of injection should be retained for as long a time as possible. The patient keeping the recumbent posture until the feeling induced is sufficient to bring about a complete emptying of the contents of the lower bowel.

A steady perseverance in the due application of the means here indicated, day by day, and week by week, for a lenghtened period, will undoubtedly ensure constant comfort and ultimately cure even in the most inveterate cases. Recent experience in my own case and practice has proved it. All aperient medicines must be eschewed; all allopathic doses discarded. They are worse than useless; they are positively injurious.

Whatever may be prescribed, homœopathically or otherwise, for this very vulgar complaint will be of little avail in these inveterate cases unless the mechanical difficulty be overcome in such a way as to bring about a natural action of the gut itself. It must be restored to its pristine condition and made capable of contracting rapidity on its contents.

This good result cannot be attained without much patience and persistent action on the part of the patient in giving full effect to the advice and injunctions herein contained.—*The Homœopathic World, London.—N. O. Med. Journal.*

**Jaborandi in Cholera.**—At the commencement of the present epidemic of cholera in Japan I determined to give, in its treatment, the still fashionable medicine jaborandi and its alkaloid a fair trial.

Though my observations are far from complete, or in all respects perfectly conclusive, I have seen enough to convince me that we have in this drug a remedy of undoubted value iu the treatment of this disease.

Thus, I find that, in the usual complete or partial suppression of urine so common in such cases, the specific effect of the medicine shows itself with almost equal frequency in exciting activity of the kidneys as of the skin and salivary glands, its administration being followed in some cases by a copious secretion of urine, with great relief to all the symptoms. When the fortunate result is not obtained in so marked a manner, the tendency to the development of uræmic symptoms is apparently less common.

When too great prostration does not exist, the copious perspiration which follows its use is attended, after the first two minutes, with a decided improvement in the character of the pulse; the sense of oppression is relieved, and a reaction often ensues which is far more natural than when produced by alcoholic or other stimulants, and with apparently less tendency to secondary fever.

A curious fact is often observed, that even when there is considerable vomiting, a drachm of the fluid extract of jaborandi, at best a nauseous dose, is retained till its specific effect is produced. In one case in which there was almost always constant vomiting, this symptom nearly ceased on my administering ¼ grain of pilocarpin hypodermically. I find, however, that this remedy has little effect in the more desperate cases, attended by perfect collapse. In fact, I am by no means sure that it does not tend to hasten the already rapidly approaching fatal termination.—*Dr. Simmons, in N. Y. Medical and Surgical Journal.*

**Iodoform.**—"All chancres are best treated with iodoform; under its use healthy sores heal rapidly, creeping sores generally cease to spread, and sluggish ones take on healthy action." My own experience supports this statement most fully. I cannot explain the manner in which it acts, but that it does have a most remarkable effect in promoting the healing, not only of ordinary chancres, but of many other sores, I can have no possible reasonable doubt. It is what I might call a *reliable* remedy, and often saves one a deal of trouble; its effects seem almost magical. You sprinkle a little of the crystals, powdered or unpowdered, over the sore, cover this with a bit of dry lint, or vaseline spread on lint, and at your inspection next day you find that healing has progressed rapidly, the sore has filled in considerably if it is a deep one, there is but little discharge and no smell, and you have only to repeat the dressing, and so go on from day to-day until the healing is complete.

Dr. Moleschott, of Turin, in the Medical Record of November, 1878: The latter speaks of its value in getttng rid of glandulous swellings, even such as occur in lyphoma and in splenic leukœmia, in orchitis, in effusion into serious cavities, in acute hydrocephalus, in chronic arthritis, in intercostal and other neuralgias, etc. He says that before promoting absorption it determines the destruction of the primitive elements, and that its power may be regarded as miraculous. He uses as an external application 1 to 15 parts of elastic collodion, or 1 to 15 parts of ointment, and advises that these preparations should be kept at a distance in the window, in a well closed tin case, thus retarding the decomposition of the iodoform, which goes on rapidly in the light. When used internally he gives three-fourths of a grain to one and a half grains daily in pills. He winds up his paper by saying, "That notwithstanding its inconveniences, I dare promise for the remedy a great future." Its inconveniences are its pungent odor and its liability occasionally to cause eructations and palpitation of the heart. —*Dr. Sheen in the Practitioner.*

**The Aspirator in Hernia.**—Dr. W. O. Roberts says in the Louisville Medical News: I reported a case of obstructed hernia relieved with the aspirator. On the evening of the 21st instant I saw, with Dr. Coleman Rogers, a similar case, which was treated successfully in the same way. The patient, a boy three years of age, had been the subject of a reducible inguinal hernia on the right side for about one year, brought on by forcible efforts at micturition, which were due to a contracted prepuce. Whenever the tumor made its appearance the mother said she would push it back, and that it would not come out again until he would have to strain in emptying his bladder. About 2 o'clock p. m., on the 21st instant, some half an hour or more after urinating, he commenced crying and pressing on his abdomen. On examination, his mother found the rupture down and much larger than usual. She attempted its reduction as upon former occasions, but without success. She then put him to bed, applied hot cloths, and continued at intervals her attempts at reduction; but failing, and the tumor steadily increasing in size and becoming very painful when handled, she took the child at 6 o'clock p. m., four hours after her attention was called to the hernia, to Dr. Rogers's office. There I saw it. The tumor was then about the size of a hen's egg, tense, and painful to the touch. The patient was quiet, excepting when the tumor was manipulated, but wore an anxious expression. The skin felt natural; pulse somewhat excited; bowels had moved twice before and once after the rupture was discovered. There had been no vomiting.

After chloroforming the patient and then failing in our attempts at reduction by manipulation, assisted by the dependent position, we tried with a hypodermic syringe, which was first tested and found to be in good order, to draw off the fluid, but did not succeed in getting a drop. Feeling certain that there *was* fluid in the sack, we procured an aspirator and with it drew off a half an ounce of straw-colored liquid, and while this was passing through the instrument—the patient being held in a perpendicular position, head downward—the tumor suddenly disappeared.

**When to Induce Premature Labor in Puerperal Eclampsia.**—The proper time to induce premature labor in puerperal eclampsia becomes at once an interesting and important question.

Will it be wise to resort to the measure early; or shall it be postponed until other means have been tried and found insufficient? While these are questions, speaking generally, which must be determined from the observation and study of each individual case, yet I

opin‿ there is more frequent occasion to regret too great delay in the employment of this method, than that it has been invoked too early.

Observation and experience, those final arbiters of all questions medical, have settled some general principles pertaining to this whole subject, from which I formate the following:

1. It is wiser to err on the side of safety to the mother, and induce labor too soon, than to temporize until it is too late. When the uterine body has attained sufficient size to interfere materially with the return blood current, the indication is most clear. This will especially be the case if there is deep coma in the interval of the fits, indicating great activity of the blood poison. Here the urgency is pressing, and prompt action may save life. In hesitation there may be disaster.

2. The amonnt of albumen in the urine is not a sure indication of the extent of the blood poison, since in many of the severer cases there are but slight traces of albumen, and in others it is wanting altogether; while, on the other hand, albuminuria is frequently found in pregnant women who do not subsequently have convulsions.

3. From this it may be inferred that the practice of inducing labor when there is albuminuria without convulsions is a questionable one; its employment as a necessity, at least, being limited to a small minority of cases.—*American Journal of Obstetrics.*

**Scilline, etc., the Active Principles of Squill.**—Squill, though deservedly placed in the first rank among diuretics, labors under the disadvantages of being uncertain in its action, owing to the difficulty of obtaining the bulbs in the proper condition, and of possessing an acro-narcotic principle which proves poisonous to some individuals, even when only moderate doses are taken. These disadvantages are now overcome, it is claimed, by Merk, of Darmstadt, who has succeeded in isolating the active principles of the bulb, and is thus enabled to exclude the poisonous principle and obtain a uniform preparation, which is adapted even for subcutaneous use.

The substance called scillitine, which has been obtained from the bulbs by extraction with alcohol, is a mixture of the three principles separated by Merk, a fact that accounts for the variability of its action. Dr. Fronmuller, of Furth, has tested Merk's three preparations of squill on fifty-three cases, and draws the following conclusions concerning them:

1. Scillitoxine, in the majority of cases, produces pretty free diuresis, but it contains the greater part of the toxic principles of the squill, and hence is not suited for clinical use.

2. Scilline also is not adapted for practical use, because it exists only in minute quantities in the bulb, is very dear, and seems to be inactive unless given in large doses.

3. Scillipicrine, dissolved in water and administered hypodermically, has proved to be a diuretic of the first class, second to none other in efficacy. Out of seventeen ca^ces of oliguria, it failed only twice. In twelve of the fifteen successful cases the quantity ol urine was doubled and often even more, and in one it was increased sixfold. No symptoms of poisoning were produced, thanks to the separation of the scillitoxine. The only disagreeable effect produced was a more or less intense irritation at the site of the injection; this was absent only in two cases, and in four it was pretty severe. In one case four tablespoonfuls per diem of a two per cent. solution of the scillipicrine were given internally without any effect. For the subcutaneous injections a ten per cent. solution was employed in most of the cases; in only one is it stated that a second injection was practiced. In two of the cases, the increased excretion of urine was accompanied by thin passages from the bowels.—*Allg. Med. Cent. Zeit.*

**External Application of the Bromide of Potassium.**—The good effects obtained from bromide of potassium in all reflex irritations due to teething are well known, but M. Peyraud claims that better results can be obtained from direct local application of the remedy to the gums, than from its internal administration. He uses a mixture of the bromide, one part, to honey six or seven parts, with sufficient water to dissolve the salt, and enough alcohol to preserve the mass. This should be gently rubbed on the gums four or five times a day; in cases of diarrhœa caused by dentition, a few drops of Sydenham's laudanum may be added with advantage. The bromide acts as an anæsthetic to the mucous membrane, as a caustic to excoriations, and through its effect on the general nervous system. It quiets immediately the urticaria of dentition, and under its influence those excessively nervous children in whom the eruption of the teeth is irregular and difficult, pass through this period without convulsive phenomena.

M. Peyraud has also used it with success in caries of the teeth and of some of the long bones, and in diphtheria. Carious teeth he fills with powdered bromide, which is retained in its place by a tampon of cotton; the first application calms the pain in about twenty minutes, and the tooth gradually becomes insensible. In caries of the long bones the fistulæ are washed out with a solution of the bromide in glycerine and water. Diphtheritic membranes are powdered with the finely pulverized salt, every two, three or four hours, according to the severity of the case. A cure results in from twenty-four hours to five days, the average being about three to four days.—*Journal de Medecine.*

**Esmarch's Tourniquet in the Treatment of Neuralgia.**—On the 24th day of August, 1879, I was treating a lady suffering with the most intense neuralgia I ever witnessed. From the lumbo-sacral region down both sciatic nerves to the feet, the paroxysms of pain were frightful, even after large and repeated hypodermic injections, including morphia sufficient to have killed her ten times over, but for the antidotal influence of pain. Every admissible remedy had been employed for forty-eight hours with scarcely any alleviation, when I thought of Esmarch's Tourniquet. I took one of Martin's No. 4 Elastic Bandages, and beginning at the toes, bandaged one limb tightly to the groin. The pain disappeared at once, but continued unabated in one limb. I took off the bandage and applied it to the other with like result. I allowed the bandages to remain about fifteen minutes on each limb. There was no return of pain. The method by which it effects a cure is obvious, and I believe that most cases of sciatica can be cured with this bandage in a much shorter time than by any other method, except two-ounce doses of turpentine, which latter I have never known to fail of cure within a space of six hours. I have tried in three cases, giving two ounces ol. terebinth in four ounces camphor water at one dose. No unpleasant symptoms, except intoxication for a few hours, and burning of the same when the bowels moved.—*Dr. Garrison in Country Pract.*

**Cholagogues.**—A commission with Dr. Rutherford, of Edinburg, at its head, has instituted an investigation into the action of certain cholagogues upon the secretion of bile. Its conclusions concerning the action of these important remedies, in daily use by the profession, are of great interest. The experiments were made upon dogs. The effects thus obtained may not be exactly similar to the action of these agents in the human system.

Podophyllin, aloes, rheum, euonymin, sanguinarin, iridin, leptandrin, colocynth, jalap, sodium phosphate, mercuric chloride, phytolaccin, hydrastin, juglandin, sodium and ammonium benzoate, benzoic acid, sodium salicylate, and nitro-muriatic acid, all are more or less powerful hepatic stimulants, positively increasing the amount of bile secreted, and all, except the last five, being more or less stimulant, and irritating to the intestinal glands. Senna, colchicum, taraxacum and scammony are feeble hepatic stimulants. Camboge. castor oil and calomel stimulate the intestinal glands, but not the liver. Ipecacuanha is simply a hepatic stimulant. Magnesium sulphate is only stimulant to the intestines, as are also ammonium chloride and menispermin.

Calabar bean stimulates the liver, and atropia antagonizes this action, but atropia administered alone does not affect the secretion of bile.

It is also found in man that four grains of iridin at night is a certain remedy for "biliousness." Euonymin, in two grain doses, also has the same effect. Both are liable to be followed by depression, if the dose be too large, and both should be followed by a saline aperient in the morning.—*British Med. Journal.*

**Hypodermic Injections of Chloral in Convulsions.**—I have great confidence in the effect of chloral and bromide potassium administered internally, but some cases resist its internal administration. One case in point is that of a little boy, aged about seven years, who had been treated in all manners and by all means till complete unconsciousness. The bowels were constipated; he had passed little urine; the skin felt clammy; the temperature was below normal; pulse slow and weak until the commencement of the paroxysm, when it increased in frequency and fullness. The attack commenced at the angles of the mouth, going to the facial muscles and those of the eye, and then to the right and left side of the body until the child was convulsed *en masse.* Realizing the inadequacy of prescribing any more of the pet prescriptions, I determined to inject four grains of hydrate of chloral hypodermically. Hardly had this been done one and a half minutes when every bad symptom disappeared, and the boy slept soundly for about four hours. He then awoke, asked for some water, and slept soundly until the next morning, experiencing no bad effects from the procedure. A small abscess at the point of insertion ensued, and was readily healed. I have tried the same method in several cases since that time, and feel some confidence in its use.—*Jos. L. Bauer, M. D., in St. Louis Clinical Record.*

**Incontinence of Urine in Children.**—At the Harveian Society of London, Dr. Farquharson read a paper on this subject. He referred to the subject under three headings :

1st. Cases observed in children of pale, weakly organizations, in which there is no doubt some weakened condition of the sphincter vesicæ, or of the nervous centres in the lumbar cord. Tonic remedies, especially small doses of wine, will act with good effect.

2. Cases of greater severity, usually dating from soon after birth, and here it is necessary to make a distinction between the enuresis by day and that by night, for the latter frequently resists all medical treatment, departing often spontaneously about the period of puberty. Belladonna, which acts upon the unstriped muscular tissue, gives the best results in such cases. It is necessary to give full doses. Ergot proved disappointing, and santonin is without influence.

Class third includes those cases in which the incontinence is truly a neurosis. Here galvanism is especially indicated, and blistering over the fifth lumbar vertebra, where modern experiment has demonstrated the motor centre to be situated. The recently proposed plan of excluding meat from the dietary was not found of much service.—*Buffalo Med. Journal.*

**Interstitial Pneumonia.**—Dr. Edwin Payne, in London Lancet, says :

In interstitial pneumonia the connective tissue of the lung is attacked. This is a very frequent disease, and if allowed to run its whole length, and come demonstrably recognizable, it is then impossible to do anything to soften or resolve the cicatricial tissue. Having in the course of both public and private practice met with such serious results in many cases, it occurred to me that prevention in such cases might be better than cure, and so when in a case the lung gives signs of persistent solidity following upon a recent attack, and does not show a tendency to recover its function upon the general restoration of power, I have noticed that iodide of potassium in full doses has had a most beneficial effect. Signs of improvement in the lung condition have followed in answer to the remedy, acting as a solvent upon a condition of structure *on its way* to be converted into a callous, bloodless substance; thus restoring structure to use instead of allowing it to arrive at a condition useful only for demonstration at a post-mortem, as the resultant of the unaided progress of diseased action.

**Subcutaneous Injection of Ergot in Neuralgia.**—Dr. Marino, of Palermo, quoted in the London Medical Record, publishes the results of his experiments with ergot: First, in tic doulourox local injections of ergot give better results than any other remedies, quinine included; second, the results are equally as good in hemicrania; third, in some cases of sciatica very good results have been obtained, while in other cases no relief has been afforded to the patient; fourth, ergot should be administered in other cases in neuralgia, especially if the latter is caused by blood poisoning or cachexia; fifth, the injection itself is often painful, but abscesses do not often supervene. The pain generally ceases in half an hour, especially if a cold compress has been immediately applied to the place; sixth, the neuralgic symptoms as a rule disappear after one or two injections, but it is advisable to continue them for some time; seventh, the dose for one injection varies from 15 centigrams to 2 decigrams of ergot, dissolved in water or glycerine.

**Mammary Inflammation Treated by Ice.**—A writer in the British Medical Journal says that the suggestion of treating threatened inflammation of the mammary gland by ice was "one of the most valuable hints he ever got." He thus describes its use in a case:

"I used a large Chapman's spine-bag, filled with ice, which encircled the lower half of the breast. It felt very cold indeed for a minute or two, then a considerable quantity of milk was shot out, as from a syringe (no milk had flowed before), the pain abated, and in an hour was almost gone; I now renewed the ice in the bag, and the patient kept it closely applied with her arm, which was protected from the cold by a folded towel. Next morning I found her hugging the ice-bag and loud in its praise. She continued suckling her infant, but she suggested that the baby should not be put to the breast oftener than two or three times in the twenty-four hours. On the fourth day after the commencement of the ice the most careful examination failed to detect anything wrong in the breast, and she is now quite well and nursing her child. No other remedies were used."—*Medical Reporter.*

**Poisoning by Carbolic Acid.**—A case of acute poisoning with carbolic acid through an enema is recorded by Dr. Praetorius in the *Berliner Klinische Wochenschrift* April 14, 1879. The patient, a delicate lady aged 45, had been suffering for several weeks from an obstinate attack of diarrhœa, which could not be stopped by the usual agents, and threatened to undermine the patient's strength. The author prescribed an enema of a one per cent. solution of carbolic acid, of which a quarter of a *litre* was mixed with one-third of a *litre* of warm water. Hardly had one-third of this enema been injected, when the patient began to complain of singing in the ears, giddiness, and weakness, and collapsed. The enema was of course immediately suspended, and the patient told to void her bowels. This was done; but she remained in a collapsed state till the bowels had been washed out with warm water, when she gradually recovered, but it was not till two hours later that the disagreeable symptoms disappeared. The diarrhœa, however, had been stopped by the carbolic acid.—*Hosp. Gazette.*

**The Choice of Purgatives.**—In amenorrhea the best are aloes and myrrh pills; in dropsies the compound jalap powder; in sciatica the compound colocynth pill, or the compound decoction of aloes; in hemorrhoids the confection of senna; in biliousness a blue piil followed up by a dose of epsom salts (the blue pill acts on the duodedum and hurries the bile downward, while the salts cause the other part of the bowel to contract, and so evacuate the bile before it is reabsorbed.) If a purgative does not act the rule should be to repeat it at once, and then, if necessary, give a copious warm water enema. From all I can see I would say the less we make use of purgatives the better. Nature knows her own work, and if we take regular mental and bodily exercise, eat and drink moderately, we shall find this as a rule quite sufficient for keeping us in good sound health, and also for preserving a *mens sana in corpore sano.—Dr. James Atkinson in Edinburgh Medical Journal.*

**Dentition as a Cause of Diarrhœa.**—Between the sixth and twenty-eighth month dentition plays a very important part in the production of diarrhœa. It might be called a nervous diarrhœa, for it is probably due to reflex nervous disturbances. If dentition is not directly responsible for many of these diarrhœas, it is indirectly so by putting the system in a condition to be more susceptible to all those influences which do produce diarrhœa. In all cases where the gums are swollen, lance them. In any case where it is about time for the tooth to come through, lance the gums over the tooth thoroughly and draw some blood. I believe the disturbance is often due to pressure of the tooth deeply in, and before it shows much swelling on the surface; lancing the gums never does harm. It is better to err on the side of lancing them when there may be no necessity; I have often seen a child having from ten to twelve movements a day relieved entirely by lancing the gums, and with no other treatment. It is in these cases that the bromide proves so effectual. Give the following combination of a bromide with mucilage to a child between six months and a year; older children a larger dose:

℞ Sodii Bromid.......................................... ʒ ss.

Mucilag. acaciæ

Aquæ puræ aa q. s. ad..............................aa ℥ ij.

M. Sig. ʒ j. q. 3 h.

The bromide diminishes the reflex disturbance, and the mucilage is soothing to the irritated intestinal mucous membrane.—*Medical Record, N. Y.*

**Dr. Robert's** list of pathological states, in which albumen occurs constantly or occasionally. (*Birmingham Medical Review*, July, 1879):

1. Acute and chronic Bright's disease of the kidneys.
2. Pregnancy and the puerperal state.
3. Febrile and inflammatory diseases (zymotic diseases, such as scarlet fever, measles, small-pox, typhoid, cholera, yellow fever, ague, diphtheria, etc.; inflammatory diseases, such as pneumonia, peritonitis, traumatic fever, acute articular rheumatism, etc.)
4. Impediments to the circulation of the blood (emphysema, heart disease, abdominal tumors, cirrhosis, etc).
5. A hydræmic and dissolved state of the blood and atony of the tissues (purpura, scurvy, pyæmia, hospital gangrene); also hæmatinuria.
6. Saturnine intoxication.
7. Nervous disturbance (neurotic albuminuria.)—*Herald.*

**A Urine Thermometer.**—Dr. Otto Kutsner, of Jena, describes an ingeniously arranged instrument composed of a silver female catheter which contains in its lumen a self-registering thermometer. The catheter tapers sharply below the point to which the bulb of the thermometer reaches, and the instrument is so arranged that the thermometer can be removed if desired.

The urine in passing out bathes the thermometer, and only fifteen seconds are required to bring the temperature up to the maximum, which is the same as that in the axilla, or within a fraction of it (0.3°–0.5° C. higher).—*N. Y. Med. Record.*

